# The application of multimodal ultrasound examination in the differential diagnosis of benign and malignant breast lesions of BI-RADS category 4

**DOI:** 10.3389/fmed.2025.1596100

**Published:** 2025-06-09

**Authors:** Chunling Li, Mumin Wu, Huang Haiqing

**Affiliations:** Cancer Hospital, College of Medicine, Shantou University, Shantou, China

**Keywords:** breast tumor, ultrasonography, shear-wave elastography, contrast-enhanced ultrasound, multimodal ultrasound examination

## Abstract

**Objectives:**

The purpose of this study was to the diagnostic value of conventional ultrasound (US), contrast-enhanced ultrasound (CEUS) and shear wave elastography (SWE) for identifying benign and malignant BI-RADS 4 breast lesions.

**Materials and methods:**

From February 2022 to November 2024, 95 patients aged 20 to 90 years with breast diseases, all of whom were female, were included. These lesions were diagnosed as BI-RADS 4 breast lesions by conventional ultrasound. All lesions were pathologically confirmed by surgical resection or tissue biopsy, and they were further evaluated by CEUS and SWE. The sensitivity, specificity, positive predictive value (PPV), negative predictive value (NPV), and accuracy of US, CEUS, and SWE were statistically analyzed, and ROC curves were generated. The diagnostic efficacy of US, US + SWE, US + CEUS, and US + CEUS + SWE were subsequently compared, with the pathology results used as the reference standard.

**Results:**

(1) Among the 95 BI-RADS 4 lesions, 44 (46.31%) were benign, and 51 (53.69%) were malignant. The sensitivity, specificity, PPV, NPV and accuracy of the BI-RADS classification via conventional US were 86.3, 72.7, 78.6, 82.1 and 80.0%, respectively. (2) The sensitivity, specificity, PPV, NPV, and accuracy of US combined with SWE in the diagnosis of breast nodules were 96.1, 79.5, 84.5, 94.6, and 88.4%, respectively. (3) The sensitivity, specificity, PPV, NPV, and accuracy of US combined with CEUS in the diagnosis of breast nodules were 84.3, 86.4, 87.8, 82.6, and 85.3%, respectively. (4) The areas under the ROC curve (AUCs) of US, US + SWE, and US + CEUS were 0.795, 0.877, and 0.917, respectively. Statistical methods were used to evaluate the US + CEUS + SWE method, and the results indicated excellent diagnostic performance. The AUC was 0.946, while the sensitivity, specificity, PPV, NPV, and accuracy were 90.7, 93.2, 94.2, 95.3, and 94.7%, respectively. In this this study, the AUCs of US, SWE, and CEUS were compared, and the results revealed that both SWE and CEUS could increase the AUC for breast lesion diagnosis with good diagnostic performance. These methods can increase the sensitivity, specificity and accuracy of the US examination when combined with conventional US. Moreover, the diagnostic performance for breast lesions was highest with the combined application of the three modalities, with a diagnostic AUC that was significantly higher than those of US alone, US + SWE and US + CEUS. The differences were significant (*p* < 0.05).

**Conclusion:**

(1) CEUS and SWE provide diagnostic information about the microvascular perfusion and tissue stiffness of lesions, respectively, which can assist in the differentiation of benign from malignant breast tumors by conventional US and improve the sensitivity, specificity and accuracy of diagnosis, especially for US BI-RADS 4a breast lesions. (2) The combined use of CEUS and SWE in conventional US enhance the overall diagnostic performance with respect to breast lesions, with the best sensitivity and specificity and the highest diagnostic efficacy. The use of US + CEUS + SWE is beneficial for further differentiating benign and malignant breast lesions according to the US BI-RADS 4, thereby reducing unnecessary biopsies or surgeries.

## 1 Introduction

According to the cancer statistics of the International Agency for Research on Cancer (IARC) in 2020, 19.3 million new cancer cases (excluding nonmelanoma skin cancer) and almost 10.0 million cancer deaths were estimated. Female breast cancer has surpassed lung cancer, as the most commonly diagnosed cancer, with an estimated 2.3 million new cases (11.7%) (1). The largest study in Brazil evaluated 5,257 breast cancer patients from a public hospital cancer registry in Barretos. In this study, 38.9% of women were diagnosed with stage II disease, and 37% were diagnosed with stage III-VI disease. Consistent with the findings in Moaes’s study, the 5-year breast cancer-specific survival rate was 95.2% for stage I patients, 87.1% for stage II patients, and 58.4% for stage III-IV patients (2, 3).

To improve diagnostic accuracy, standardize ultrasound descriptions, and facilitate communication among physicians, the American College of Radiology (ACR) has developed and released the Breast Imaging Reporting and Data System (BI-RADS), which aims to standardize the assessment of breast lesions. According to the white paper in the ACR BI-RADS atlas, a breast lesion is assigned to a category after the US features of the lesion are evaluated. Among them, BI-RADS category 4 (BI-RADS 4) is defined as a suspicious malignancy with a malignancy probability ranging from 2 to 95% according to the BI-RADS subcategory. Specifically, BI-RADS 4a (low suspicion), 4b (intermediate suspicion) and 4c (moderate suspicion) have associated malignancy probabilities of 2–10%, 10–50% and 50–95%, respectively (4).

Traditional breast imaging methods include digital mammography (DM), ultrasonography (US) and magnetic resonance imaging (MRI). According to the literature, the sensitivity of mammography for dense breasts ranges from 62 to 68%, which is too low to ensure adequate detection of malignant breast lesions (5, 6). When B-mode ultrasonography is used alone, the positive predictive values (PPVs) and 95% confidence intervals (CIs) are 13.6% (95% CI: 11, 16%) for BI-RADS 4a, 50.0% (95% CI: 44, 56%) for BI-RADS 4b and 86.0% (95% CI: 82, 90%) for BI-RADS 4c (7). Notably, when the two-dimensional ultrasound imaging features of some breast lesions overlap on conventional ultrasound images, distinguishing between benign and malignant lesions becomes difficult. With respect to MRI, although the resolution is high for soft tissues, the MRI technology is complex, the procedure is time-consuming, there are many contraindications, and the examination is expensive. Furthermore, the imaging characteristics of breast lesions cannot be truly observed by MRI. Moreover, specialists who can perform supplementary mammography or conventional ultrasound examinations for difficult cases are lacking.

Ultrasonic elastography and contrast-enhanced ultrasound are common methods used for differentiating benign from malignant breast lesions by means of ultrasound. Research shows that the combination of ultrasonic elastography and microblood perfusion features can improve the diagnostic performance for identifying malignant and benign breast lesions. By integrating conventional ultrasound examination, SWE and CEUS, information on the blood flow and hardness of the breast lesions can be obtained. This method can be used to evaluate the ultrasound imaging characteristics of breast tumors further, thus assisting radiologists in better differentiating benign from malignant breast tumors (8, 9). In this article, we cite the relevant content from this literature: The breast cancer diagnosis technique is operator-dependent and demands the skills of a veteran diagnostician. Nevertheless, many influences such as fatigue and a lack of attentiveness can be a source of misdetection, leading to low survival rates. To counteract this, CAD techniques have been proposed and evaluated, but they are challenging to implement due to the variety of cells, structure, quality of the image, and resemblance among benign and malignant trials (10–13). Therefore, the aim of this study was to explore the diagnostic efficacy of SWE, CEUS, and the combination of SWE and CEUS in conventional ultrasound to differentiate benign from malignant breast lesions. We also investigated whether this can improve the sensitivity, specificity, and accuracy of the BI-RADS 4 diagnosis, with pathology results used as the reference standard. The broader goal is to better apply and promote these methods to future ultrasound-based diagnoses of breast lesions.

## 2 Materials and methods

### 2.1 Patients

This retrospective single-center study was approved by the local institutional review board and included 95 breast lesions (age range20–90 years; mean 52.82 ± 3.47 years) between February 2022 to November 2024. All participants provided written, informed consent. The inclusion criteria were as follows: (1) focal breast lesions detected by conventional ultrasound examination, with no treatment or intervention; (2) conventional ultrasound diagnosis of the BI-RADS classification into one of the 4 categories (including 4a, 4b, and 4c); and (3) available biopsy and/or pathology diagnosis results.

The exclusion criteria were as follows: (1) pregnant or lactating; (2) maximum lesion diameter exceeding the sampling frame of contrast-enhanced ultrasound and elastography by ultrasound; (3) non-mass breast lesions were difficult to visualize on ultrasound; (4) superficial location of the lesion, with the anterior edge of the lesion being less than 3 mm from the anterior edge of the breast skin or very deep, with the anterior edge of the lesion being more than 4 cm from the anterior edge of the breast skin; (5) poor-quality US images; (6) there is no evaluation of the surgical and pathological response.(7) there is no surgical specimen.

**Figure 1:**
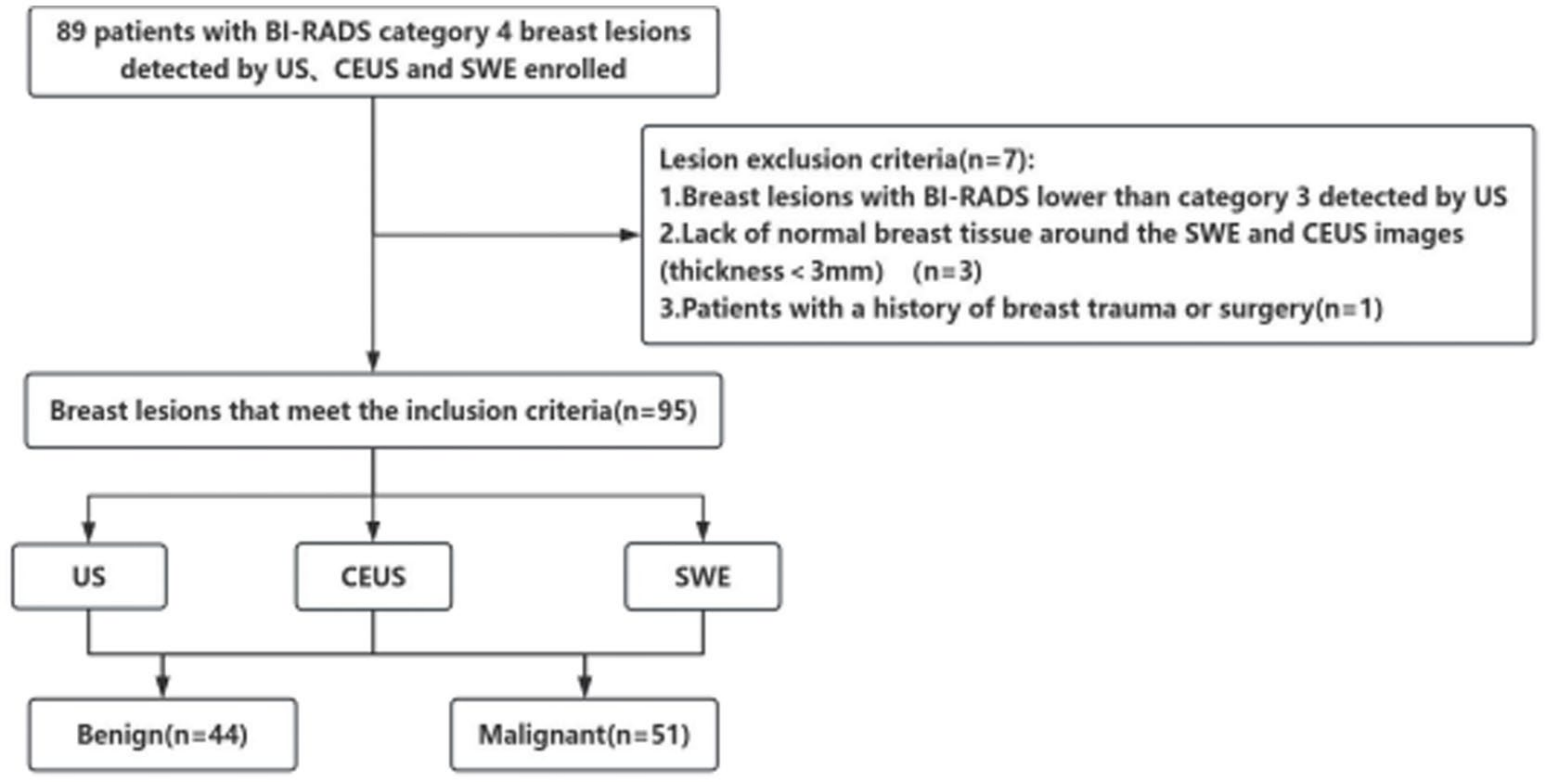
Flowchart showing selection of patients.

Prior to surgical excision, all subjects underwent conventional US, CEUS and SWE examinations. The pathological results of the samples obtained by surgery or biopsy were regarded as the reference standard. The interval between the histopathological examination and the ultrasound examination was less than 1 week (Figure 1). Before the pathological diagnosis is confirmed, the patient is unaware of whether they are classified under the BI-RADS grading system. During the study, all patients were required to undergo US, CEUS and SWE examinations before pathological biopsy. Our ultrasound physicians made diagnoses of the lesions according to the 2013 ACR BI-RADS classification criteria (14). After these examinations, the pathological results of the surgery were used to verify the ultrasound examination results. Patients with a history of breast surgery were excluded from the study. In addition, informed consent forms were obtained from each patient before enrollment.

### 2.2 US examination and pathological assessment

In this study, Mindray Resona R9 ultrasound diagnostic equipment equipped with L15-3WU linear array probes (frequency 3–15 MHz) and L9-3U linear array probes (frequency 3–9 MHz), as well as Mindray Resona 7 ultrasonic diagnostic equipment equipped with L14-5 linear array probes (frequency 5–14 MHz) and L9-3 linear array probes (frequency 3–9 MHz), was used for conventional two-dimensional ultrasound examination, shear-wave ultrasound elastography, and contrast-enhanced ultrasound. All these devices were equipped with SWE and CEUS analysis software. The contrast agent used for ultrasound was sulfonylhexafluoride (SonoVue), 59 mg/vial, which was produced by Bracco, Italy. Five milliliters of normal saline was added to prepare a microbubble suspension for intravenous injection. In the patient evaluation stage, two ultrasound physicians with more than 10 years of experience in ultrasound diagnosis recorded and analyzed the basic characteristics of the breast lesions by means of the ultrasound examination according to the 2013 ACR BI-RADS classification criteria and finally obtained the diagnostic results of the lesions (14). If there was a disagreement, a consensus was reached through discussion or by consulting a third experienced ultrasound physician. In addition, the designated examining physicians will hold academic communication every week to report on the enrollment situation, ensuring the smooth progress of the study and the accuracy of the data. During the implementation of the study, we will strictly standardize the research protocol to ensure consistency across different research sites. Specific measures include: uniformly using the same model of ultrasound equipment to reduce the impact of equipment differences on the results; the fixed examining physicians will have academic communication every week. Besides reporting the enrollment status, they will also randomly select several examination results for re-evaluation. In terms of data entry, we stipulate that the data should be directly entered into the system by the fixed physicians who conduct the examinations, so as to reduce data errors that may be caused by personnel changes.

SWE measurements were carried out according to the reference standard of ultrasound elastography established by the results of Mindray Medical’s multicenter study, and the measurements were conducted as follows: elastography uses the maximum elastic modulus value (Emax) within a 2-mm area around the breast lesion (shell area, E) as the test variable. An Emax value greater than 98.66 kPa indicated malignancy, whereas an Emax value less than 98.66 kPa indicated a benign lesion. The positive indicators for CEUS are as follows: ① When the peak of enhancement is reached, the lesion shows high enhancement; ② the lesion shows heterogeneous enhancement or is accompanied by perfusion defects; ③ the lesion starts to enhance earlier than the surrounding breast tissue; ④ after enhancement, the range of the enhanced area of the lesion expands compared with that of two-dimensional ultrasound; ⑤ after enhancement, the edge of the lesion appears irregular or spiculated, and shows crab-claw-like enhancement; and ⑥ radial or tortuous blood vessels can be seen inside or around the lesion. A score of ≥4 indicates a diagnosis of a malignant lesion, whereas a score of ≤ 3 indicates a diagnosis of a benign lesion (15).

The pathological diagnosis was made by pathologists with over 10 years of working experience who were unaware of the ultrasound and other imaging results. The pathological features of the breast lesions, including tumor type, size (measured via gross pathology), histological grade, vascular invasion status, and lymph node status, were recorded. The tumors were classified according to the 7th edition of the breast TNM staging system.

### 2.3 Statistical analysis

All data were analyzed using SPSS 27.0 software. For continuous variables, normality tests were first conducted. Data that followed a normal distribution were presented as mean ± standard deviation (x ± s), and independent sample *t*-tests were used for comparison between groups. Data that did not follow a normal distribution were presented as median (inter-quartile range), and intergroup comparisons were performed using the Mann–Whitney *U* test. Categorical variables were described by frequencies and percentages. According to the sample size and theoretical frequency, the *χ*^2^test or Fisher’s exact probability method was selected for the comparison of differences between groups. *p*<0.05 was considered statistically significant.

In this study, we determined the values of TP (True Positives), FP (False Positives), TN (True Negatives), and FN (False Negatives) by comparing the diagnostic results of various ultrasound examinations with the pathological diagnosis results, which served as the gold standard. Subsequently, we utilized the following formulas for further calculations: Sensitivity = TP / (TP + FN); Specificity = TN / (TN + FP); Positive Predictive Value (PPV) = TP / (TP + FP); Negative Predictive Value (NPV) = TN / (TN + FN); Accuracy = (TP + TN) / (TP + TN + FP + FN). The prediction model was built via the logistic equation. A receiver operating characteristic (ROC) curve was constructed to assess the diagnostic performance of the prediction model, and the area under the ROC curve (AUC), sensitivity, specificity, accuracy, positive predictive value (PPV) and negative predictive value (NPV) were calculated, with pathology results used as the reference standard. The ROC curve analysis was conducted to assess the diagnostic performance of US, SWE, CEUS, US +SWE, US + CEUS and US + SWE + CEUS, and a *Z* test was conducted to compare the AUC values. Categorical data are presented as *n* (%) and were compared by means of the chi-square test. A *p* value < 0.05 was considered statistically significant. ROC plots were constructed for individual and combined diagnoses with Medcalc15.0 software.

## 3 Results

### 3.1 Histopathologic diagnosis

In total, 95 breast lesions were included in this study for the final analysis, among which 83 were solitary lesions and 6 were multiple lesions. The maximum diameter of the lesions ranged from 5.10 to 52.50 mm, with an average size of 20.72 ± 2.81 mm. Among the 95 lesions, 46.3% (44/95) were benign, and 53.7% (51/95) were malignant (Table 1). The mean ages of patients with benign and malignant breast lesions were 48.61 ± 2.95 years and 56.45 ± 3.52 years, respectively. The mean maximum diameters of the benign and malignant breast lesions were 18.50 ± 2.34 mm and 22.63 ± 2.63 mm, respectively. There was no significant difference in age or lesion size between patients with benign tumors and those with malignant tumors (*p* > 0.05).

**Table 1:** Histopathological results.

Benign lesions	Number	Malignant lesions	Number
Fibroadenoma	26	Infiltrating ductal carcinoma	45
Adenosis of breast	5	Intraductal carcinoma	5
Benign phyllodes tumor	7	Mucinous carcinoma	1
Intraductal papilloma	3		
Foreign - body granuloma	1		
Plasma cell mastitis	2		
Total	44	Total	51

### 3.2 The diagnostic value of SWE and CEUS in differentiating benign from malignant BI-RADS 4 lesions

The quantitative and qualitative analysis parameters of SWE and contrast-enhanced ultrasound for differentiating benign and malignant breast lesions classified as BI-RADS 4 are shown (in Tables 2, 3). With respect to SWE, in the differential diagnosis of benign and malignant breast lesions, significant differences were observed in color composition, color uniformity, Tozaki score, hard ring sign, Emax, and Emax-shell-2 mm. The malignant lesions were mainly green and orange/red, accounting for 45.10 and 41.18%, respectively, whereas blue was the main color among the benign lesions (68.18%). These findings indicate that color composition is significant in the differential diagnosis of benign and malignant lesions (*p* < 0.001). In terms of color homogeneity, only 9.80% of the malignant lesions were homogeneous, 62.75% were moderately uniform, and 27.45% were non-uniform; in the benign lesions, 47.73% of the benign lesions were homogeneous. These findings suggest that malignant lesions tend to have poor color uniformity, and this characteristic can assist in differentiation (*p* < 0.001). Both Tozaki scores of 3–4 points and the stiff rim sign are indicative of malignant lesions, accounting for 82.35 and 92.16%, respectively. The average values of these two indicators for malignant lesions were 140.01 kPa and 174.21 kPa, respectively, which were significantly higher than those for benign lesions (70.02 kPa and 82.27 kPa). These values can serve as a reference for differentiating benign and malignant lesions (*p* < 0.001) (Table 2).

**Table 2:** Comparison of SWE characteristics between benign and malignant breast lesions.

SWE	Breast masses		*p*	Diagnostic threshold
	Malignant (*n* = 51)	Benign (*n* = 44)		
Color composition				
Predominantly Blue	7 (13.73)	30 (68.18)	<0.001	Predominantly Green
Predominantly Green	23 (45.10)	11 (25.00)		
Orange/Red	21 (41.18)	3 (6.82)		
Color uniformity				
Uniform	5 (9.80)	21 (47.73)	<0.001	Somewhat Non-uniform
Somewhat Non-uniform	32 (62.75)	22 (50.00)		
Non-uniform	14 (27.45)	1 (2.27)		
Tozaki				
1–2	9 (17.65)	35 (79.55)	<0.001	3–4 分
3–4	42 (82.35)	9 (20.45)		
Hard rim sign				
Present	47 (92.16)	8 (18.18)	<0.001	Present
Absent	4 (7.84)	36 (81.82)		
Emax/kPa	140.01 (126.44, 157.50)	70.02 (61.57, 83.85)	<0.001	85.07
Emax-shell-2 mm/kPa	174.21 (162.57, 194.72)	82.27 (72.50, 100.07)	<0.001	99.06

**Table 3:** Comparison of CEUS characteristics between benign and malignant breast lesions.

CEUS	Breast masses		*p*	Kappa	p*
	Malignant (*n* = 51)	Benign (*n* = 44)			
The starting time of mass enhancement					
Faster	45 (88.24)	16 (36.36)	<0.001	0.527	<0.001
Equal /later	6 (11.76)	28 (63.64)			
The degree of mass enhancement at the peak					
Marked hyperenhancement	41 (80.39)	13 (29.55)	<0.001	0.511	<0.001
Equal/low enhancement	10 (19.61)	31 (70.45)			
enhancement shape					
Regular	8 (15.69)	41 (93.18)	<0.001	0.773	<0.001
Irregular	43 (84.31)	3 (6.28)			
Boundary of enhancement					
Clear	8 (15.69)	37 (84.09)	<0.001	0.678	<0.001
Fuzzy	43 (84.31)	7 (15.19)			
Direction of contrast agent perfusion					
Centripetal	5 (9.80)	4 (9.09)	>0.001	0.007	>0.001
Non-centripetal	46 (90.20)	40 (90.91)			
Uniformity of contrast agent distribution					
Uniform	5 (9.80)	29 (65.91)	<0.001	0.547	<0.001
Non-uniform	46 (90.20)	15 (34.09)			
Marginal nourishing vessels					
Absent	30 (58.82)	41 (93.18)	<0.005	0.355	<0.0055
Present	21 (41.18)	3 (6.82)			
Contrast agent perfusion defect					
Absent	13 (25.49)	34 (77.27)	<0.001	0.515	<0.001
Present	38 (74.51)	10 (22.73)			
The range increased after enhancement					
Absent	7 (13.73)	39 (88.64)	<0.001	0.793	<0.001
Present	37 (72.55)	2 (4.55)			
Indistinguishable	7 (13.73)	3 (6.82)			
Contrast agent retention in venous phase					
Absent	11 (21.57)	41 (93.18)	<0.001	0.717	<0.001
Present	40 (78.43)	3 (6.82)			

Interobserver consistency was assessed using the Kappa test; *p-value corresponding to the Kappa test.

Contrast-enhanced ultrasound (CEUS) revealed significant differences in the enhancement time, peak enhancement degree, enhancement shape, enhancement boundary, contrast agent distribution homogeneity and presence of peripheral feeding vessels between benign and malignant breast lesions classified as BI-RADS 4. In terms of the enhancement onset time of the masses, 88.24% of the malignant lesions were enhanced more rapidly than the benign lesions were. Among the malignant lesions, 80.39% showed significantly high peak enhancement, with 84.31% exhibiting an irregular enhancement shape and 84.31% having a blurred boundary. Compared with benign lesions, these characteristics have significant reference value for judgment (*p* < 0.001) (Table 3).

**Figure 2:**
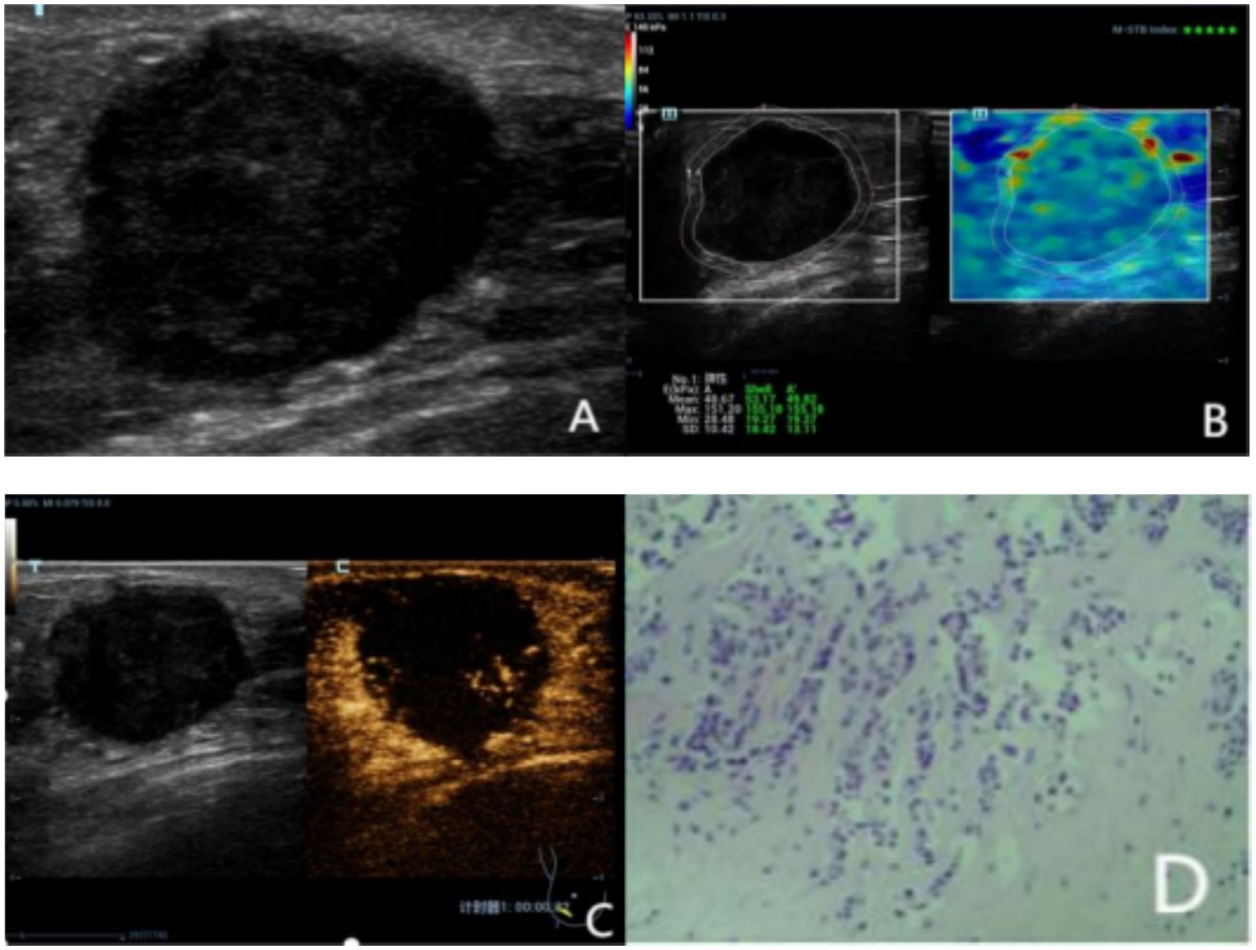
A 72 year-old female was diagnosed with Invasive carcinoma with histopathology. **(A)** The conventional US image indicated a hypoechoic area at 7 o’clock direction of the right breast with ill-defined margins, 40 mm from the nipple, with a size of 25 × 21 × 26 mm and its margin with slight lobulation. It was categorized as BI-RADS 4a. **(B)** Shear wave elastography (SWE) shows that the elasticity value of the Shell 2 mm region was 155.18 kPa, which is judged as malignant. After combining with conventional ultrasound (US), the BI-RADS category was upgraded by one level to 4b. **(C)** Contrast - enhanced ultrasound (CEUS) reveals that during the arterial phase, the nodule enhances faster than the surrounding glands, showing heterogeneous enhancement with local non - enhancement. After enhancement, the nodule has an irregular shape, an enlarged range, and feeding vessels around it. The CEUS score is 5 points, indicating a malignant diagnosis. After combining with conventional US, the BI-RADS category is upgraded by one level to 4b. After SWE + CEUS combined with conventional US for diagnosis, the BI-RADS category is upgraded by two levels to 4c. **(D)** Histopathological analysis revealed invasive carcinoma (hematoxylineosin stain; original magnification, ×100).

**Figure 3:**
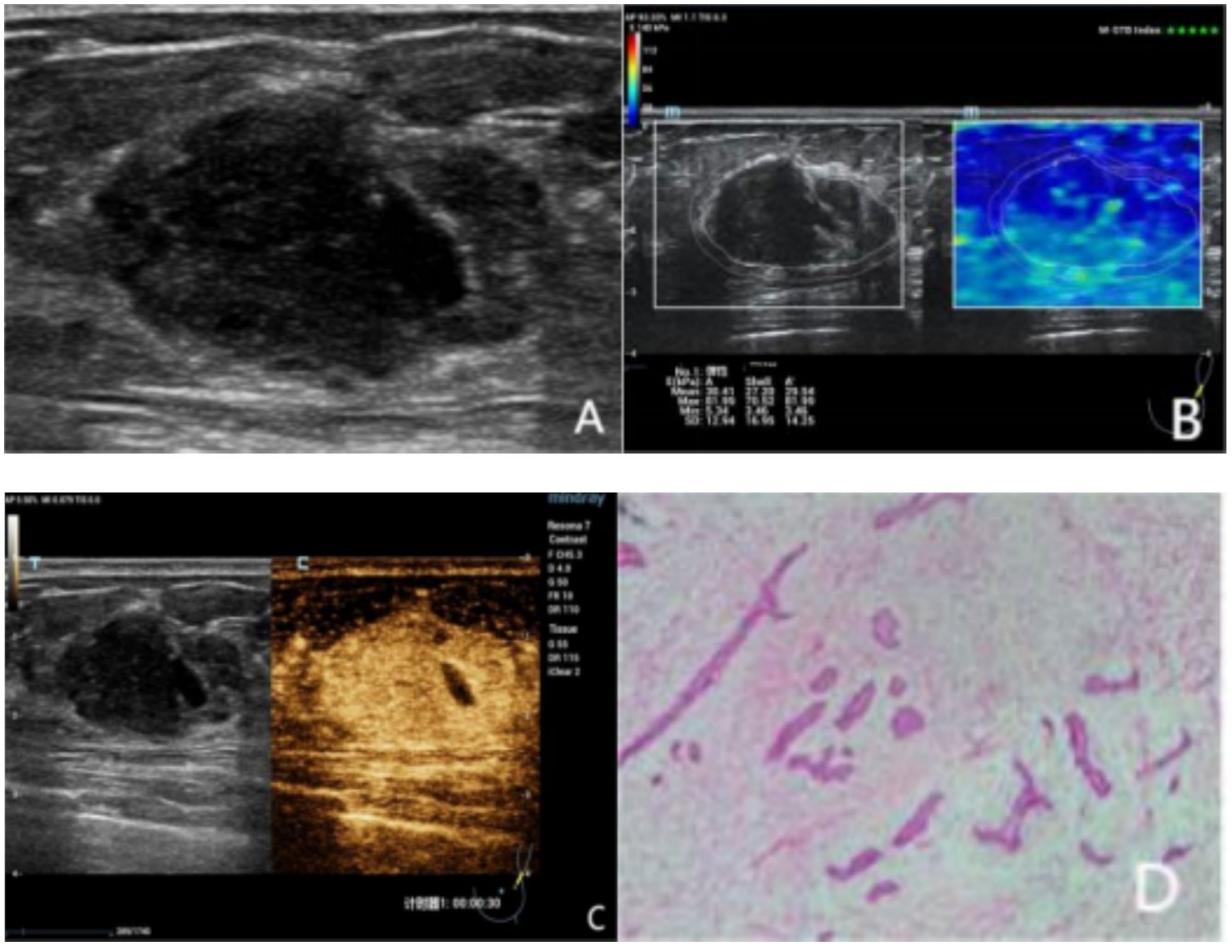
A 60 year-old female was diagnosed with a mass detected in the left breast. **(A)** The conventional US image indicated a hypoechoic area at 2 o’clock direction of the left breast with ill-defined margins, with a size of 30 × 20 × 23 mm and it has an irregular shape, unsmooth edge, lobulated. It was categorized as BI-RADS 4b. **(B)** Shear wave elastography (SWE) shows that the elasticity value of the Shell 2 mm region was 70.52 kPa, which is judged as benign. After combining with conventional ultrasound (US), the BI-RADS category was upgraded by one level to 4a. **(C)** Contrast - enhanced ultrasound (CEUS) reveals that during the arterial phase, the nodule enhances almost simultaneously with the surrounding glandular tissue, showing homogeneous enhancement. After enhancement, the shape of the nodule is clear and regular, the enhanced range does not expand, and there are feeding vessels around it and feeding vessels around it. The CEUS score is 3 points, indicating a benign diagnosis. After combining with conventional US, the BI-RADS category is upgraded by one level to 4a. After SWE + CEUS combined with conventional US for diagnosis, the BI-RADS category is upgraded by two levels to 3 category **(D)** Histopathological analysis revealed benign phyllodes tumor of the breast (hematoxylineosin stain; original magnification,×100).

However, there was no significant difference in the direction of contrast agent perfusion between benign and malignant lesions (*p* > 0.001). Among the malignant lesions, 90.20% had an uneven distribution of contrast agent, 41.18% had peripheral feeding vessels, 74.51% had contrast agent perfusion defects, 72.55% had an enlarged range after enhancement, and 78.43% had contrast agent retention in the venous phase. In contrast, the proportions in these aspects were opposite for benign lesions (Figures 2, 3).

### 3.3 Comparison of diagnostic performance of different methods

**Figure 4:**
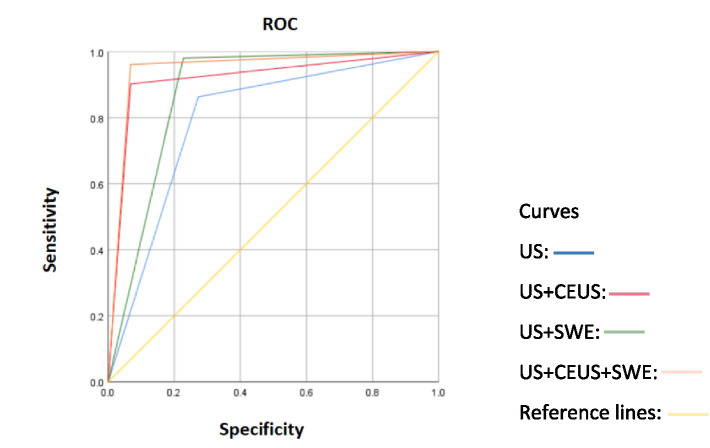
ROC curves of different diagnostic methods combined with conventional US for diagnosing benign and malignant breast lesions.

The sensitivity, specificity, PPV, NPV and accuracy of this multimodal diagnostic method for distinguishing between benign and malignant breast tumors are summarized in Table 4. Compared with those on conventional US, some relevant parameters for diagnosing benign and malignant breast tumors noticeably improved on US + SWE, US + CEUS and US + SWE + CEUS all, with the accuracy gradually increasing. The highest detection accuracy of 94.7% was achieved by US + SWE + CEUS. The AUC of US + SWE + CEUS was considerably higher than that of US (0.946 vs. 0.795, *p* < 0.05) and US +SWE (0.946 vs. 0.877, *p* < 0.05). Furthermore, the AUC of US + SWE + CEUS was also greater than that of US +CEUS (0.946 vs. 0.917), but the difference was not significant. (Figure 4). Thus, the diagnostic efficacy of both US + SWE + CEUS and US + CEUS was better than that of conventional ultrasound for breast tumors (Table 4).

**Table 4:** Comparison of diagnostic efficacy of multiple combined diagnostic methods for benign and malignant breast tumors.

Diagnostic method	AUC	95% CI	Sensitivity	Specificity	PPV	NPV	Accuracy
US	0.795	0.700–0.89 0	86.3	72.7	78.6	82.1	80.0
SWE	0.899	0.830–0.96 8	96.1	79.5	84.5	94.6	88.4
CEUS	0.878	0.800–0.95 6	84.3	86.4	87.8	82.6	85.3
US+SWE	0.877	0.798–0.95 6	98.0	79.5	84.7	97.2	89.5
US+CEUS	0.917	0.853–0.98 1	88	95.5	95.7	87.5	91.6
US+SWE + CEUS	0.946	0.893–1.00 0	90.7	93.2	94.2	95.3	94.7

US, ultrasound; CEUS, contrast-enhanced ultrasound; SWE, shear wave elastography.

### 3.4 Comparison of US, SWE, and CEUS characteristics of breast lesions in different age groups

**Figure 5:**
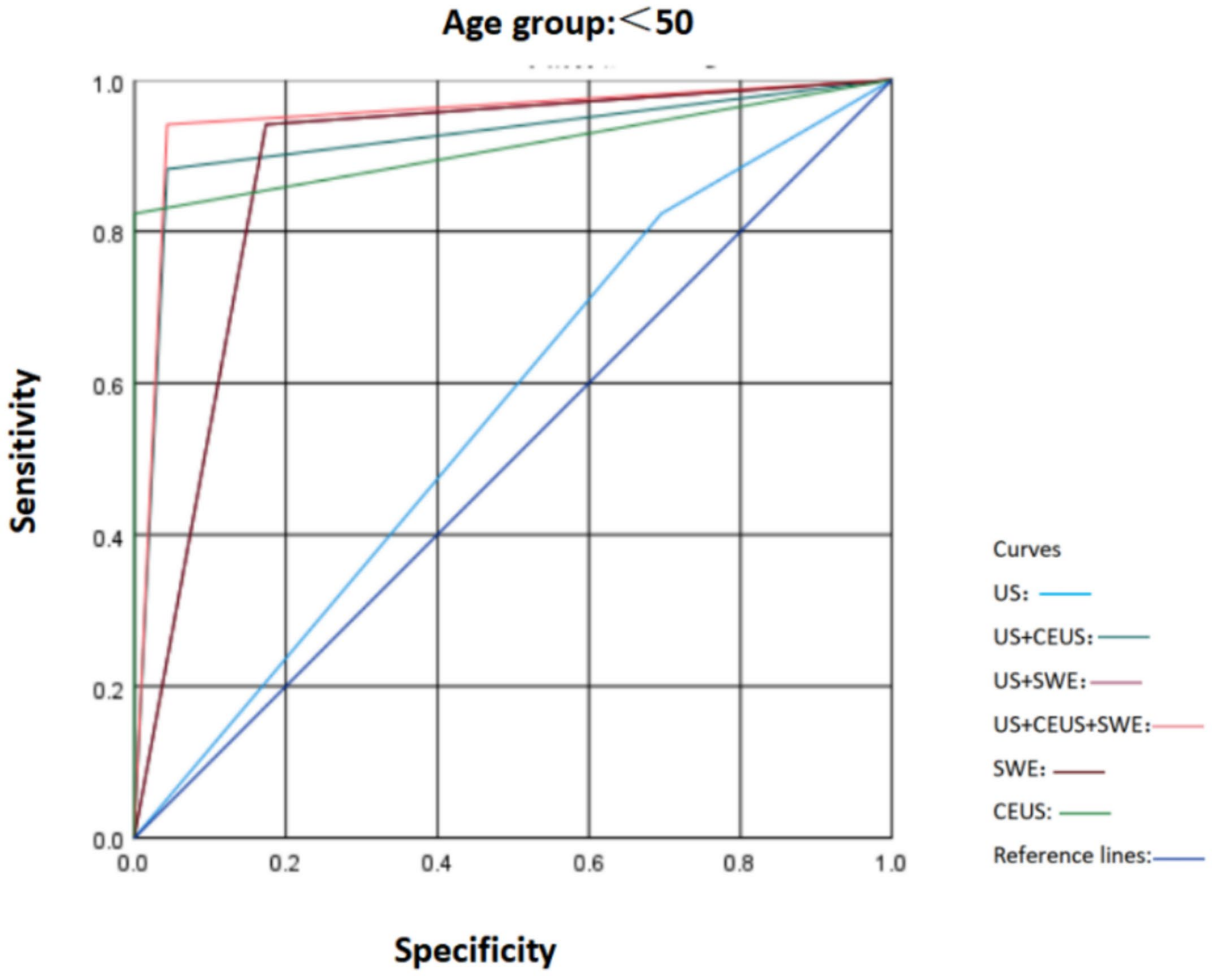
Comparison of ROC curves of US, SWE, and CEUS and other ultrasound diagnostic methods for breast lesions in the age group < 50.

**Figure 6:**
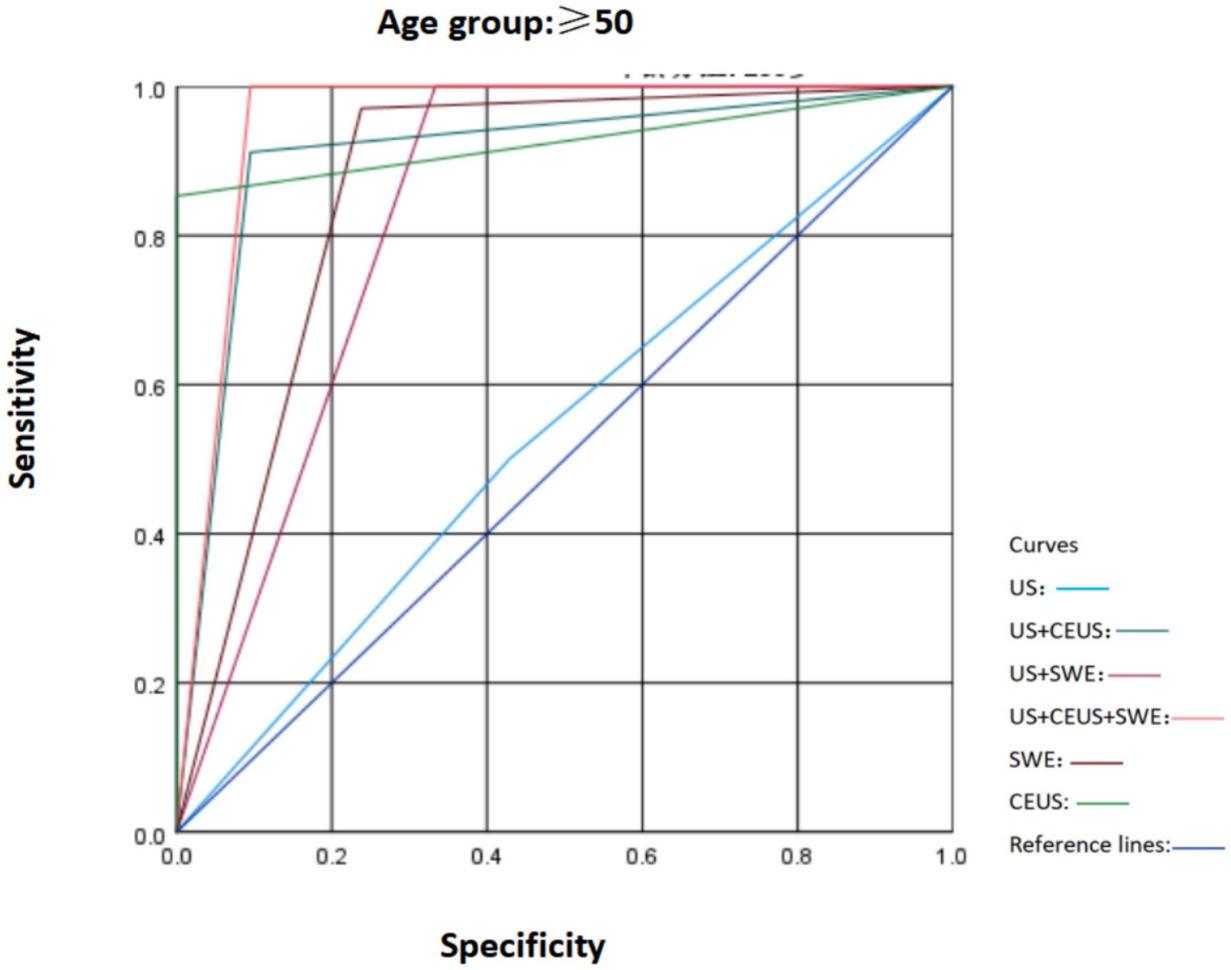
Comparison of ROC curves of US, SWE, and CEUS and other ultrasound diagnostic methods for breast lesions in the age group ≥ 50.

To ascertain the robustness of the expanded cohort study results and in accordance with the literature, according to the most recent Surveillance, Epidemiology, and End Results Program (National Cancer Institute, USA) data, breast cancer incidence is 42.84/100,000 per year in women younger than 50 and 338.55/100,000 per year in women older than 50. In our study population, the prevalence of malignant tumors in women under 50 years old is 0.05%, indicating that the likelihood of women under 50 developing cancer is extremely low (16). Therefore, based on the original study, we divided the patients’ ages into those over 50 years old and those under 50 years old. We compared the characteristics of SWE, US, and CEUS in different age groups and plotted the ROC curves (Figures 5, 6). It was found that in the population under 50 years old, the combined diagnosis of CEUS and SWE had the highest diagnostic efficacy (AUC = 0.949). When used alone, the diagnostic efficacy of CEUS and SWE was better than that of US, and the combined diagnosis could significantly improve specificity and accuracy. In the population of 50 years old and above, the combined diagnosis of CEUS and SWE had the same and highest diagnostic efficacy (AUC = 0.952) as the combined diagnosis of CEUS. When used alone, CEUS has a relatively higher diagnostic efficacy compared to US and SWE. The combined diagnosis could ensure high sensitivity while improving specificity and accuracy (Tables 5–8).

**Table 5:** Comparison of US characteristics among different age groups [*n* (%)].

US	Age		Statistic	*p*
	Less than 50 years old (*n* = 40)	50 years old and above (*n* = 55)		
Ultrasonic measurement size	19.5 (15, 26)	20 (15, 26)	−0.396	0.692
Solid				
Cystic - solid	1 (2.5%)	1 (1.8%)	0.052	1.000
Solid	39 (97.5%)	54 (98.2%)		
Echogenicity				
Hypoechoic/Markedly hypoechoic	29 (72.5%)	43 (78.2%)	1.874	0.392
Isoechoic	8 (20.0%)	11 (20.0%)		
Mixed echogenicity	3 (7.5%)	1 (1.8%)		
Calcification				
Absent	28 (70%)	34 (61.8%)	6.166	0.046
Coarse	3 (7.5%)	0 (0%)		
Microcalcification	9 (22.5%)	21 (38.2%)		
Aspect ratio				
<1	33 (82.5%)	33 (60.0%)	5.258	0.024
>1	7 (17.5%)	22 (40.0%)		
Margin				
Clear/Smooth lobulation	12 (30.0%)	16 (29.1%)	0.009	1.000
Obscured/Coarse lobulation	28 (70.0%)	39 (70.9%)		

**Table 6:** Comparison of SWE characteristics among different age groups [*n* (%)].

SWE	Age		Statistic	*p*
	Less than 50 years old (*n* = 40)	50 years old and above (*n* = 55)		
Color composition				
Predominantly Blue	15 (37.5%)	22 (40.0%)	0.096	0.953
Predominantly Green	15 (37.5%)	19 (34.5%)		
Orange/Red	10 (25.0%)	14 (25.5%)		
Color uniformity				
Uniform	11 (27.5%)	15 (27.3%)	0.033	0.984
Somewhat Non-uniform	23 (57.5%)	31 (56.4%)		
Non-uniform	6 (15.0%)	9 (16.4%)		
Tozaki				
1–2	19 (47.5%)	25 (45.5%)	0.039	0.844
3–4	21 (52.5%)	30 (54.5%)		
Hard rim sign				
Present	23 (57.5%)	32 (58.2%)	0.004	1.000
Absent	17 (42.5%)	23 (41.8%)		
Emax/kPa	84.69 (63.39, 137.10)	109.53 (73.22, 147.71)	−1.236	0.216
Emax-shell-2 mm/kPa	97.09 (69.71, 188.09)	149.09 (81.33, 190.11)	−1.221	0.222

**Table 7:** Comparison of CEUS characteristics among different age groups [*n* (%)].

CEUS	Age		Statistic	*p*
	Less than 50 years old (*n* = 40)	50 years old and above (*n* = 55)		
The starting time of mass enhancement				
Faster	23 (57.5%)	38 (69.1%)	1.354	0.283
Equal /later	17 (42.5%)	17 (30.9%)		
The degree of mass enhancement at the peak				
Marked hyperenhancement	21 (52.5%)	33 (60.0%)	0.531	0.532
Equal/low enhancement	19 (47.5%)	22 (40.0%)		
Enhancement shape				
Regular	23 (57.5%)	27 (49.1%)	0.657	0.533
Irregular	17 (42.5%)	28 (50.9%)		
Boundary of enhancement				
Clear	23 (57.5%)	22 (40.0%)	2.845	0.101
Fuzzy	17 (42.5%)	33 (60.0%)		
Direction of contrast agent perfusion				
Centripetal	5 (12.5%)	4 (7.3%)	0.738	0.486
Non-centripetal	35 (87.5%)	51 (92.7%)		
Uniformity of contrast agent distribution				
Uniform	14 (35.0%)	20 (36.4%)	0.019	0.891
Non-uniform	26 (65.0%)	35 (63.6%)		
Marginal nourishing vessels				
Absent	33 (82.5%)	38 (69.1%)	2.205	0.158
Present	7 (17.5%)	17 (30.9%)		
Contrast agent perfusion defect				
Absent	22 (55.0%)	25 (45.5%)	0.844	0.409
Present	18 (45.0%)	30 (54.5%)		
The range increased after enhancement				
Absent	21 (52.5%)	25 (45.5%)	4.032	0.133
Present	14 (35.0%)	28 (50.9%)		
Indistinguishable	5 (12.5%)	2 (3.6%)		
Contrast agent retention in venous phase				
Absent	24 (60.0%)	27 (49.1%)	1.108	0.307
Present	16 (40.0%)	28 (50.9%)		

**Table 8:** Comparison of diagnostic efficacy of combined diagnostic methods for breast tumor benignity and malignancy among different age groups.

Age	Diagnostic method	AUC	95% CI	Sensitivity	Specificity	PPV	NPV	Accuracy
<50	US	0.564	0.384–0.744	82.4	30.4	46.7	70.0	56.4
	US+CEUS	0.919	0.817–1.000	88.2	95.7	93.8	91.6	91.9
	US+SWE	0.884	0.770–0.997	94.1	82.6	80.0	95.0	88.4
	US+SWE + CEUS	0.949	0.867–1.000	94.1	95.7	94.2	95.6	94.9
	SWE	0.884	0.770–0.997	94.1	82.6	80.0	95.0	88.4
	CEUS	0.912	0.801–1.000	82.4	100.0	100.0	88.5	91.2
≥50	US	0.536	0.378–0.694	50.0	57.1	65.3	41.4	53.6
	US+CEUS	0.908	0.816–1.000	91.2	90.5	94.0	86.4	90.8
	US+SWE	0.833	0.705–0.962	100.0	66.7	82.9	100.0	83.3
	US+SWE + CEUS	0.952	0.878–1.000	100.0	90.5	94.5	100.0	95.2
	SWE	0.866	0.750–0.982	97.1	76.2	86.8	94.2	86.6
	CEUS	0.926	0.853–1.000	85.3	100.0	100.0	80.8	92.6

US, ultrasound; CEUS, contrast-enhanced ultrasound; SWE, shear wave elastography.

## 4 Discussion

Breast cancer is the most common malignancy among women worldwide. Ultrasound has long been an important tool for the widespread screening of breast lesions in China. According to the 2013 version of the ACR BI-RADS guidelines, the likelihood of cancer for BI-RADS 3 is clearly stated as >0% to≤2% (14). The probability of cancer for BI-RADS category 4 is greater than 2% and less than 95% and the probability of cancer for BI-RADS category 5 is clearly stated to be greater than or equal to 95% (14). In the early stage, we conducted an in-depth analyses of some cases classified as BI-RADS 3 and BI-RADS 5, and obtained several important findings. This indicates that in BI-RADS 3, the degree of malignancy is relatively low. Additionally, combining CEUS and SWE technologies has limited effect on reducing the positive predictive value (PPV), and it will increase the examination costs for patients. In cases of BI-RADS category 5, it highly suggests a great possibility of malignancy. In this situations, whether using US alone or in combination with CEUS and SWE for examination has a relatively limited role in clarifying the nature of the lesion, because the malignant tendency of these cases is already quite obvious. And according to the results of our statistical analysis of BI-RADS 3 and BI-RADS 5, there is no statistically significant difference. The cases of BI-RADS 4 causing difficulties for some patients in making clinical treatment decisions. Comprehensively considered from the perspective of economic cost-effectiveness, cases of BI-RADS 4 have unique value. In this category, our research method, which is to combine CEUS and SWE with routine US examination, can fully play its role, minimizing unnecessary biopsies and accurately differentiating the benign and malignant nature of the lesions. According to the literature, BI-RADS category 4a refers to low suspicion of malignancy (>2–10% likelihood of malignancy), category 4b refers to a moderate suspicion of malignancy (>10–50% likelihood of malignancy), and category 4c refers to a high suspicion of malignancy (>50% but <95% likelihood of malignancy) (17). This means that the wide range of malignant risk probabilities for BI-RADS 4 lesions is related to the high variability of breast cancer. Ultrasonography (US) was used for 186 BI-RADS 4 nonpalpable breast lesions whose known diagnoses were reviewed retrospectively; the lesions were malignant in 38.7% of cases and benign in 61.2% of cases. This situation may lead to an increase in unnecessary biopsies (18).

The early diagnosis of breast cancer through screening mammography (MG) has been proven to significantly reduce mortality but is subject to significant limitations including modest sensitivity, which gives rise to a 30–50% interval cancer rate, as well as a low positive predictive value (PPV), leading to a large burden of follow-up and many breast biopsies. Dense breast tissue, which is encountered in over 50% of women, further decreases the sensitivity and specificity of mammography (19–21). Ultrasound examination is an important method for screening and diagnosing breast diseases. However, the manifestations of conventional grayscale images of breast lesions in BI-RADS category 4 and BI-RADS category 3 often overlap to some extent and are sometimes difficult to distinguish. This may lead to a relatively high false-positive rate and consequently result in unnecessary biopsies and treatments (22–24). Ultrasound is a non-invasive, low-cost technique that does not use ionizing radiation and provides a “real-time” image, and for these reasons, this method is ideal in several situations (25). Ultrasound (US) is used for both diagnosis and supplemental screening but is also associated with a low PPV and leads to further increased cost and additional biopsy recommendations (19, 26). Some reports indicate that in current practice, up to 80% of breast lesions that undergo biopsy turn out to be benign. As a result, the number of breast biopsies has skyrocketed, with some estimates suggesting that the annual rate of these procedures in the United States alone may be as high as 1,000,000 per year (27, 28). This places a high economic burden on society, estimated to be USD 2 billion annually (29, 30), and is emotionally burdensome to patients.

In recent years, with the development of medical technology, CEUS and SWE have been added as supplementary methods. By combining their advantages, diagnostic information such as the morphology of lesions, blood flow characteristics, and hardness inside and around the lesions can be obtained. This increases diagnostic accuracy and provides new possibilities for differentiating benign lesions from malignant breast lesions. CEUS can display the microcirculation perfusion and anatomical morphological features of lesions and surrounding tissues in real time, such as the quantity, shape, and spatial distribution of new blood vessels. Moreover, it can indirectly reflect hemodynamic characteristics through the enhancement pattern, which gives it a unique advantage in differentiating between benign and malignant lesions (31). Compared with traditional ultrasound, CEUS is less dependent on the operator (32). Most malignant lesions showed typical heterogeneous enhancement which is due to the expanding, tortuous, irregular thick and thin, and irregular-shape of the neovascularization, sometimes accompanied with luminal stenosis and blocked. In contrast, the angiogenesis of the benign tumor that shows normal proliferation and thickening, and have the same size and homogenous distribution. Meanwhile, the vessels of malignant lesion are repaired and randomly distributed, presenting irregular shape, irregular thickness, tortuous, and forming arteriovenous fistula or vessel caecum for thrombus (33–35). In this study, benign tumors such as fibroadenoma and intraductal papilloma were examined by CEUS imaging alone, and the capsule or pseudocapsule was clear, showing isoechogenic enhancement, hypoechogenic enhancement, and uniform enhancement, and there was no increase in size after the contrast-enhanced examination. Malignant tumors have no capsule due to infiltrative growth, have feeding vessels and liquid necrosis. In the early stage, there is centrifugal heterogeneous hyperenhancement. There were six false positive cases and eight false negative cases in the examination results. Among the false positive cases, there were two cases of adenosis of the breast and two cases of intraductal papilloma, with the contrast-enhanced manifestations showing an expanded enhanced area and unclear margins. In one case of benign phyllodes tumor, the contrast-enhanced manifestation was an expanded enhanced area. In one case of plasma cell mastitis, pyogenic inflammation occurred in the center of the lesion, resulting in a filling defect in the center during the contrast-enhanced examination, while the periphery showed irregular hyperenhancement, and the enhanced area expanded after the contrast-enhanced examination. Currently, although many studies on the ultrasonographic features of breast cancer using CEUS exist, the lack of a unified diagnostic standard has restricted its wide application in the diagnosis of breast diseases.

Shear wave elastography (SWE) is an ultrasound-based imaging method that can be used to obtain the histological information of lesions of interest by means of the mechanical index (elasticity) of tissues. The hardness of lesion tissues can be quantitatively assessed by measuring the propagation speed of shear waves, thereby reducing the need for breast biopsies (36, 37). Previous studies have shown that rather than the center of the malignant lesion, the periphery of malignant breast lesions is the hardest. Increasing attention has been given to the surrounding areas of breast lesions, especially the hardness of the outer shell (38). Research has shown that, when a shell-based analysis method is used, the Emax of the shell at 2.0 mm has the highest diagnostic performance in differentiating benign from malignant breast tumors (39, 40). Our study revealed that the AUC, sensitivity, specificity, and accuracy of SWE for diagnosis were 89.9, 96.1, 79.5, and 88.4%, respectively. However, there may be some overlap in the elasticity coefficients of tissues under different conditions. If the lesion is too deep (the distance from the leading edge of the lesion to the anterior edge of the breast skin is ≥4 cm) or if the lesion contains liquid components, or even internal calcification, it may lead to diagnostic bias in SWE. In the cases of this study, only shear wave elastography (SWE) imaging was used for examination. The examination results showed 9 false positive cases and 2 false negative cases. The pathological types of the false positive cases were fibroadenoma, benign phyllodes tumor, and foreign body granuloma, respectively. This may be related to the increase in the tissue hardness value of the lesions caused by the presence of tissue fibrosis, sclerosis, or calcification within the lesions. The diagnostic performance of SWE alone for differentiating benign and malignant breast lesions was significantly better than that of conventional US. However, when US is combined with SWE, the main missed malignant lesions are those in the low-invasion categories, such as invasive ductal carcinoma, intraductal carcinoma, mucinous carcinoma and other types of tumors. This might be because the tumors had necrosis, hemorrhage, or a soft texture, leading to reduced tissue stiffness. When studying elastography, the distance between the lesion and the nipple, as well as the depth of the lesion, can cause uneven pressure on the lesion and stress attenuation, which may affect strain and shear wave elastography. Compared with the use of SWE and CEUS alone to assist conventional US, the combination of SWE and CEUS can reduce the limitations of breast nodule examination. This combined approach can more comprehensively reflect the biological and microcirculatory characteristics of breast lesions. It achieves higher sensitivity, specificity, and accuracy, thereby effectively enhancing the ability to differentiate between benign and malignant breast lesions. At the same time, this combined application can compensate for the lack of experience of young physicians, reduce subjective differences, lower the missed diagnosis rate, help detect lesions at an early stage, and reduce the mortality rate of patients. Therefore, the combined diagnosis of CEUS + SWE may help patients avoid unnecessary biopsies for BI-RADS category 4 lesions that do not require clinical intervention. Meanwhile, it does not delay the optimal treatment time for malignant lesions. It is an ideal non-invasive clinical diagnostic method and is worthy of promotion and application in clinical practice.

In this study, it was found that if both SWE and CEUS are positive, the likelihood of the lesion being malignant is higher; if both SWE and CEUS are negative, the lesion is more likely to be benign; if SWE is positive and CEUS is negative, the lesion is more likely to be benign. The situation where SWE is negative and CEUS is positive is rare. However, both CEUS and SWE are based on two-dimensional grayscale ultrasound, which usually requires careful observation of the lesion’s ultrasound features, such as the aspect ratio, margin, echo pattern and the presence of internal microcalcifications, in addition to considering the clinical history. In current clinical practice, ultrasound physicians cannot do without these important imaging features and medical history information. However, if we use SWE and CEUS simultaneously to assist conventional US, we can significantly improve the diagnostic sensitivity and specificity for breast masses.

There was no statistically significant difference in various characteristics of CEUS and SWE among different age groups (*p* > 0.05), indicating that age is not the main factor influencing the characteristics of CEUS and SWE of breast tumors. Age only affects some conventional ultrasound characteristics of breast tumors. Among them, the proportions of microcalcifications and an aspect ratio greater than 1 were higher in women aged 50 and above (*p* = 0.046 and *p* = 0.024, respectively), which may be related to the higher possibility of cancer in women aged 50 and above (16). In terms of diagnostic efficacy: In the group under 50 years old, the combined use of CEUS and SWE had the highest diagnostic efficacy (AUC = 0.949); in the group aged 50 and above, the combined use of CEUS and SWE also had the highest diagnostic efficacy (AUC = 0.952). In both age groups, the diagnostic efficacy of conventional ultrasound (US) alone was relatively low, while the combination of CEUS and SWE could significantly improve the diagnostic efficacy for the benign and malignant nature of breast tumors in different age groups.

In this study, the diagnostic performance of US + SWE + CEUS for breast lesions was superior to that of US alone, US + SWE, and US + CEUS, with an AUC of 94.6% and an accuracy of 94.7%. Compared with those of SWE or CEUS alone to assist conventional US, the sensitivity, specificity, positive predictive value, and negative predictive value of US + SWE + CEUS were greater, all exceeding 90%. Women with BI-RADS 4 breast masses can benefit from the combination of SWE and CEUS, reducing unnecessary biopsies without reducing the detection rate of breast cancer.

## 5 Conclusion

CEUS and SWE provide diagnostic information regarding the microvascular perfusion and tissue hardness of lesions, respectively. The addition of CEUS and SWE to conventional US improves its diagnostic sensitivity, specificity and accuracy. This is beneficial for further differentiation of benign and malignant lesions in US BI-RADS 4 cases, thus reducing unnecessary biopsies or surgeries. Further studies with larger sample sizes and higher sensitivity are needed to minimize false-negative results and confirm the reliability of this method in clinical settings.

## Data Availability

The original contributions presented in the study are included in the article/supplementary material, further inquiries can be directed to the corresponding author.
